# Patient and Clinician Perspectives on Expanding Telehealth Use for Older Adults Across the Cancer Control Continuum: Mixed Methods Study

**DOI:** 10.2196/73058

**Published:** 2026-02-09

**Authors:** Robin T Higashi, Bella Etingen, Jessica Lee, Suzanne Cole, John C Mansour, Alice Zhao, Timothy P Hogan

**Affiliations:** 1 Department of Social and Behavioral Sciences, O'Donnell School of Public Health, The University of Texas Southwestern Medical Center, 5323 Harry Hines Blvd, Dallas, TX, 75390-9066, United States, 1 214-648-3645; 2 Harold C. Simmons Comprehensive Cancer Center, Dallas, TX, United States; 3 Research and Development Service, Dallas VA Medical Center, Dallas, TX, United States; 4 Department of Health Economics, Systems, and Policy, O'Donnell School of Public Health, The University of Texas Southwestern Medical Center, Dallas, TX, United States; 5 School of Medicine, The University of Texas Southwestern Medical Center, Dallas, TX, United States; 6 Division of Hematology and Oncology, Department of Internal Medicine, The University of Texas Southwestern Medical Center, Dallas, TX, United States; 7 Department of Surgery, The University of Texas Southwestern Medical Center, Dallas, TX, United States; 8 Center for Health Optimization and Implementation Research (CHOIR), VA Bedford Healthcare System, Bedford, MA, United States

**Keywords:** telemedicine, video visits, remote technology, older adults, remote cancer care, cancer prevention, cancer treatment

## Abstract

**Background:**

Reliance on telehealth increased dramatically during the COVID-19 pandemic, introducing new opportunities to consider the use of telehealth across the cancer control continuum. However, patient, clinician, and staff perspectives about the types of cancer care appointments that are considered appropriate and the clinical care needs to support expanded remote care services are limited. Understanding older adults’ diverse technology needs and perspectives is especially important given that they comprise a large and growing proportion of patients with cancer.

**Objective:**

This study aimed to describe the perceptions and experiences of older patients with cancer and their clinical care team members regarding the expansion of telehealth use across the cancer control continuum and to solicit suggestions about how to support telehealth use for cancer care delivery.

**Methods:**

Using a convergent mixed methods design, we surveyed and interviewed patients aged ≥60 years, clinicians, and staff at a comprehensive cancer center in the southern United States between December 2020 and November 2021. Interview questions were rooted in the sociotechnical model, which proposes 8 interrelated dimensions representing factors influencing the design, use, and outcomes associated with health information technologies. Patient survey domains included telehealth experience and satisfaction and factors affecting telehealth perceptions and use; clinician survey domains included contexts of telehealth appropriateness, training, and barriers and facilitators to telehealth service provision. Survey data were analyzed using descriptive statistics. Qualitative data were thematically analyzed using a combined deductive and inductive approach.

**Results:**

We received completed surveys from 128 patients (567 invited) and 106 clinicians and staff (146 invited). We completed 14 patient (29 invited) and 20 clinician and staff (22 invited) interviews. Across all participants, most agreed or strongly agreed that multiple cancer care appointment types should be offered via telehealth, including discussing treatment side effects (75/102, 73.5% of patients and 66/94, 70.2% of clinicians and staff), results communication (71/102, 69.6% of patients and 65/94, 69.1% of clinicians and staff), and treatment follow-up (67/102, 65.7% of patients and 52/93, 55.9% of clinicians and staff). In interviews, participants elaborated on factors influencing the appropriateness of telehealth versus in-person appointments, including symptom severity, type of cancer, and purpose of the appointment. Many patient and staff suggestions focused on ways to address digital literacy gaps, while clinicians recommended improving clinic workflows, infrastructure, and training.

**Conclusions:**

Overall, clinicians, staff, and older patients with cancer all responded positively toward expanding telehealth use across multiple cancer and appointment types across the cancer control continuum. Older adults with cancer are generally interested in telehealth for cancer care, especially if strategies to address digital literacy gaps are incorporated. Clinicians and staff members expressed specialized training and infrastructure needs to optimize telehealth uptake and service delivery.

## Introduction

**Figure 1.**
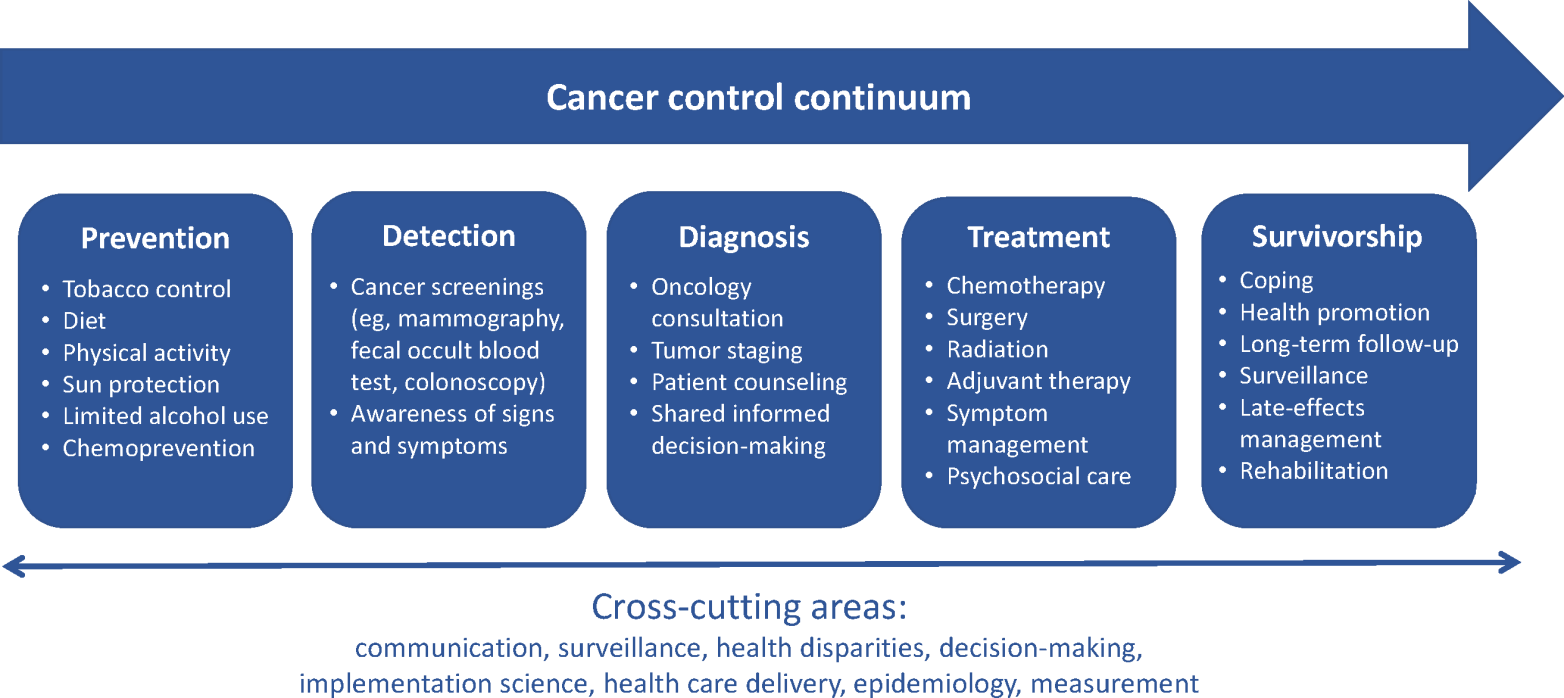
Cancer control continuum, adapted from the National Cancer Institute [17].

Synchronous video visits, or telehealth, have transformed the way cancer care is delivered, accelerated in large part due to the COVID-19 pandemic [1-4]. Avoiding infection due to COVID-19 was particularly important for patients with cancer, who were twice as likely to contract the SARS-CoV-2 virus and 8 times as likely to die from it compared to individuals without cancer [5,6]. In the pre-COVID era, telehealth services for patients with cancer primarily focused on the survivorship phase of the cancer control continuum [7-10] (see Figure 1), addressing issues like long-term monitoring and rehabilitation [11,12]. Among cancer survivors, telehealth had been shown to increase independence, convenience, quality of life, and patient satisfaction with care [13-16]. However, patient and clinician attitudes toward broader implementation of telehealth across other phases of the cancer control continuum, detection, diagnosis, and treatment, for example [17,18] are limited.

During the COVID pandemic, health care systems rapidly developed and deployed new remote care protocols and processes for patients across the cancer control continuum, with little time to assess patient and clinician needs and preferences. Cancer centers reported between 33% and 50% of patient visits occurring via telehealth, compared to rates formerly in the single digits [19]. Given that approximately 65% of cancer cases occur in patients aged 65 years and older [20,21], as well as the potential for this age demographic to face disparities in technology use, we had anticipated the need to examine the impact of the rapid expansion of telehealth on older patients specifically. Before the pandemic, a growing body of literature had begun to assess digital health literacy and technology preferences among older adults. Attitudes were diverse and multiple factors affected inequities in health literacy in addition to age, including socioeconomic status, level of education, and rural residence, particularly in the delivery of health care services [22-27]. The transition to telehealth use beyond the survivorship phase created an opportunity to understand shareholder perspectives on both the types of appointments and clinical needs that would be appropriate for remote care, and what could be done to bridge the digital health disparities facing older adults.

The objective of this study was to explore patients’ and clinicians’ pandemic-era experiences and perceptions of the appropriateness of telehealth expansion for different encounter types across the cancer control continuum. We also solicited suggestions for enhancing access to telehealth in the current era of increasing remote cancer care delivery.

## Methods

### Study Design

Using a convergent mixed methods design [28], we conducted surveys and semistructured interviews with patients, clinicians, and staff at a National Cancer Institute-designated comprehensive cancer center (“Cancer Center”) located at a large academic medical center in the southern United States. We conducted a small number of interviews as a preliminary step to inform the question items and domains included on subsequent survey instruments and to capture participant perceptions and experiences in more depth than could be captured through surveys alone. Concurrently, we used surveys to measure the robustness of experiences and perceptions with a larger sample than could be accomplished in interviews.

### Instrument Design

We selected survey domains by drawing on the literature and the sociotechnical model [29], which proposes 8 interrelated dimensions that represent the range of factors that can influence the design, use, and outcomes associated with health information technologies. The patient survey addressed a range of domains relevant to perceptions of and experiences with telehealth, including access and use, challenges encountered, perceived usefulness, and satisfaction. The clinician and staff survey similarly addressed a range of domains, including extent of experience with telehealth, perceived appropriateness of telehealth in different clinical contexts, training, and barriers and facilitators to use. In all, the patient survey comprised 89 unique questions and the clinician staff survey 124 unique questions, including Likert scale ratings, categorical items, and open-ended responses. We pilot tested the survey and interview guides with clients, caregivers of patients with cancer, and staff members of a local community-based organization for seniors in 4 focus groups prior to launching data collection. Their insights helped us optimize the content, wording, and length of instruments.

In addition to exploring some of the survey domains in greater depth, interview guides for clinicians and staff reflected their unique roles (eg, patient care, administrative leadership, and front-line clinical and administrative staff). Additionally, interviews with patients, clinicians, and staff included questions soliciting suggestions about how to improve telehealth services and the role of telehealth in cancer care in the future.

### Sampling

#### Surveys

We sampled patients for surveys from the Adult Cancer Patient Registry at the Cancer Center (n=4037) (Multimedia Appendix 1). Eligible patients were ≥60 years of age, had completed at least one telehealth encounter since March 15, 2020 (approximately the start of the COVID-19 pandemic), were English- or Spanish-speaking, were open to contact for research through the portal, and were diagnosed and received treatment for breast, prostate, colorectal, and/or hematological cancer (in order to achieve a diverse sample by cancer type; n=2464). Of these individuals, we identified n=580 patients for survey recruitment, first stratified by cancer organ site in proportion with the Adult Cancer Patient Registry, then randomly selected within each organ category by date of telehealth appointment, age, gender, and race and ethnicity to achieve a diverse sample. We invited all employees across the Cancer Center to participate in the electronic survey.

#### Interviews

Patients recruited for semistructured interviews followed the same eligibility criteria as those for the survey but additionally were identified as being in active cancer treatment (defined as receiving chemotherapy, radiation, or being within 6 months postsurgery) at the time of recruitment. We also purposefully recruited 3 patients for interviews who met all criteria except that they had not completed a telehealth encounter in an effort to understand possible reasons for nonengagement or barriers to telehealth.

The primary investigators (RTH and TPH), who are nonphysicians with expertise in qualitative methods, worked with Cancer Center leaders and clinical co-investigators (SC and JM) to create a purposive sample of clinicians and staff given the diversity of cancer care services available. We jointly identified individuals with expertise across various organ sites (breast, colorectal, prostate, and heme), roles (medical oncologists, surgical oncologists, radiation oncologists, clinical staff, and administrative staff), and levels of telehealth proficiency.

### Recruitment

#### Surveys

Because our objective was to survey patients who had completed telehealth appointments, and because telehealth appointments require the use of a patient portal account at the Cancer Center where we conducted our work, we first contacted patients through a messaging function within the patient portal with an invitation to complete the survey. Patients who did not respond to the portal message within one week were sent the same message to their personal email on file in the electronic health record. Those who did not respond to the first email inviting them to complete the survey, or who did not decline to participate, were sent a reminder email one week later, and those who did not respond to both emails within 2 weeks were sent an opt-out invitation letter by postal mail before they were called twice and offered the option to complete the survey by telephone, online through REDCap (Research Electronic Data Capture; Vanderbilt University; RRID:SCR_003445), or on paper via postal mail.

We recruited Cancer Center clinicians and staff via email for both surveys and interviews. The survey invitation included a link to take the survey in REDCap [30]. Clinicians and staff invited to participate in interviews were provided the option to schedule the interview via video conferencing or telephone. Two additional emails were sent if a survey response was not logged or if no reply was received to the interview invitation.

#### Interviews

Patients who completed a survey and who indicated in the survey a willingness to be contacted for interview participation were in turn recruited for interviews. We conducted semistructured telephone interviews with 14 patients, 11 of whom completed at least one telehealth appointment since March 15, 2020; we also purposefully recruited 3 patients who had never completed a telehealth appointment to provide contrasting perspectives. Patients who had completed telehealth appointments were recruited the same way as survey participants. The 3 patients who did not complete a telehealth appointment were mailed an opt-out letter, then contacted by phone. Up to two attempts were made to contact each of these participants.

### Data Collection

#### Surveys

We conducted patient surveys between July and October 2021 and clinician and staff surveys between April and June 2021. Patient surveys took approximately 20 minutes to complete, while clinician and staff surveys took approximately 10 minutes to complete.

#### Interviews

We conducted patient interviews between August and November 2021 and clinician and staff interviews between December 2020 and September 2021. Patient interviews lasted 24‐78 minutes (average 51 minutes) and were conducted via telephone in English or Spanish by bilingual, qualitatively trained nonphysician research staff (RTH and Ana Belen Conrado). Clinician and staff interviews lasted 29‐54 minutes (average 41 minutes) and were conducted via telephone or videoconferencing per individual preference by nonphysician investigators (RTH and TPH). Interviewers recorded brief field notes following each interview, which were reviewed and discussed in weekly meetings with members of the research team to determine when thematic saturation had been reached and thus data collection ended.

### Data Analysis

Survey data were analyzed using descriptive statistics. Audio-recorded interviews were transcribed by a professional vendor and data were managed using NVivo 12.0 (QSR International; RRID:SCR_014802). Interviews were analyzed using a thematic content analysis approach. Two qualitatively trained researchers (RTH) first developed an initial, deductively driven codebook corresponding to the semistructured interview guide domains and sociotechnical model. They jointly coded the first 20% of transcripts, sampling from a diverse set of clinician and staff roles to refine codebook definitions and add and define codes as needed based on emergent findings. The codebook was then finalized, and researchers independently coded the remaining 80% of transcripts, meeting weekly to resolve discrepancies. The lead qualitative analyst (RTH) then reviewed thematic code reports and selected representative quotes and findings for discussion and interpretation with the research team. In line with our mixed methods design, to assess areas of convergence between the survey and interview findings, the team used a joint display approach [31] to systematically integrate findings from the interviews corresponding to survey questions that we had chosen to explore in greater depth (eg, preferences for appointment type, perceptions of the telehealth implementation process, and training or preparedness for telehealth use).

We engaged the Cancer Center leaders and clinical co-investigators in 2 small group meetings to review the data and discuss their interpretations of the findings.

### Ethical Considerations

This study was approved by the Institutional Review Board at the University of Texas Southwestern Medical Center. All participants provided verbal informed consent to participate in interviews in accordance with the approved protocol (STU 2020‐0919). Participants who completed the survey received a $10 gift card. All patients who completed an interview received a $20 gift card. Clinicians and staff did not receive remuneration per institutional policy. Consent included recognition that identifiable information about participants would not be included in any publications or reports resulting from the research.

## Results

### Overview

Of 567 patients and 146 clinicians and staff invited to participate in the survey, 128 patients (23% recruitment rate) and 106 clinicians and staff (73% recruitment rate) completed the survey. For interviews, we contacted 29 patients who completed the survey and had indicated they were willing to be contacted for interviews; 3 people were unavailable after the maximum 3 attempted calls, 12 declined to participate (“not interested after all”), and 14 patients consented and were interviewed. We invited 22 clinicians and staff to participate in interviews; 2 did not respond, and 20 consented and participated in an interview. Demographic characteristics of survey and interview participants are shown in Table 1.

The qualitative and quantitative findings converged across multiple domains. In this analysis, we focus on two principal thematic findings: (1) agreement in patients’ and clinicians’ openness toward expanding cancer care via telehealth beyond the survivorship phase of the cancer control continuum, and (2) comparison between patients’ and clinicians’ recommendations about how to enhance telehealth in the future. Representative quotes from semistructured interviews are documented by theme in Multimedia Appendix 2.

**Table 1.** Demographics of patient and clinician and staff survey and interview participants.

Total	Patient surveys (n=131), n (%)	Clinician and staff surveys (n=106), n (%)	Patient interviews (n=14), n (%)	Clinician and staff interviews (n=20), n (%)
Gender				
Women	55 (42)	57 (54)	7 (50)	10 (50)
Men	61 (47)	14 (13)	7 (50)	10 (50)
No response	15 (11)	35 (33)	0 (0)	0 (0)
Age (years)				
60‐69	55 (42)	—^a^	—^b^	—^a^
70‐79	50 (38)	—^a^	—^b^	—^a^
≥80	12 (9)	—^a^	—^b^	—^a^
≤35	—^a^	23 (22)	—^b^	2 (10)
36‐45	—^a^	25 (24)	—^b^	7 (35)
46‐55	—^a^	11 (10)	—^b^	4 (20)
56‐65	—^a^	11 (10)	—^b^	3 (15)
≥66	—^a^	2 (2)	—^b^	3 (15)
No response	14 (11)	34 (32)	—^b^	1 (5)
Race or ethnicity				
Asian	3 (2)	8 (8)	0 (0)	4 (20)
Black	10 (8)	4 (4)	2 (14)	1 (5)
Hispanic	9 (7)	11 (10)	0 (0)	2 (10)
White	93 (71)	43 (41)	12 (86)	12 (60)
Two or more race or ethnicity	2 (2)	3 (3)	0 (0)	1 (5)
Other	0 (0)	1 (1)	0 (0)	0 (0)
No response	14 (11)	36 (34)	0 (0)	0 (0)
Language				
English	117 (89)	106 (100)	14 (100)	20 (100)
Spanish	1 (1)	0 (0)	0 (0)	0 (0)
No response	13 (10)	0 (0)	0 (0)	0 (0)
Driving time from home to Cancer Center				
<15 minutes	4 (3)	—^a^	—^a^	—^a^
15‐30 minutes	38 (29)	—^a^	—^a^	—^a^
31‐59 minutes	49 (37)	—^a^	—^a^	—^a^
1‐2 hours	18 (14)	—^a^	—^a^	—^a^
>2 hours	8 (6)	—^a^	—^a^	—^a^
No response	14 (11)	—^a^	—^a^	—^a^
Role				
Administrative managers	—^a^	3 (3)	—^a^	3 (15)
Administrative staff (eg, Medical Office Assistant)	—^a^	8 (8)	—^a^	2 (10)
Clinical staff (eg, Clinical Staff Assistant, Dietician)	—^a^	6 (6)	—^a^	1 (5)
Clinicians (eg, MD, NP, PA, Psychologist)	—^a^	26 (25)	—^a^	13 (65)
Nurse (RN, LVN)	—^a^	32 (30)	—^a^	1 (5)
No response	—^a^	31 (29)	—^a^	0 (0)

a Not applicable.

b Not available.

### Theme #1: Perceptions Toward Expanding Telehealth to Other Phases of the Cancer Control Continuum

#### Patients

Both patient and clinician and staff participants responded positively toward questions about the future use of telehealth during other phases of the cancer control continuum. A majority of surveyed patients indicated they would be “willing to have a video visit with a member of their cancer care team” for discussing treatment side effects (75/102, 73.5%), results communication (71/102, 69.6%), and treatment follow-up (67/102, 65.7%), as shown in Table 2. Of note, less than 6% (6/102) of our sample indicated that telehealth should not be used for any types of cancer care appointments.

**Table 2.** Patient perceptions regarding appointment types for which telehealth should be offered (n=102).

Appointment type (check all that apply)	Value, n (%)
Discussing treatment side effects	75 (73.5)
Results communication	71 (69.6)
Treatment follow-up	67 (65.7)
New treatment planning	48 (47.1)
Routine check-up	32 (31.4)
Planning the end of cancer treatment	32 (31.4)
Postoperative surgical follow-up	28 (27.5)
Genetic counseling	26 (25.5)
Physical evaluation	13 (12.7)
None	6 (5.9)
Other appointments	3 (2.9)
Total	102 (100)

In semistructured interviews, patients cited various reasons why they would feel comfortable having certain types of appointments via telehealth versus in-person, including how they were feeling physically, whether the appointment was routine, whether they had extensive questions, or whether the appointment was with a new clinician. Patients also mentioned that, regardless of appointment type, telehealth was often a convenient option for purely logistical reasons. Some had small children, others had mobility impairments, and still others noted that an easy public transportation system was unavailable to them.

#### Clinicians and Staff

**Figure 2.**
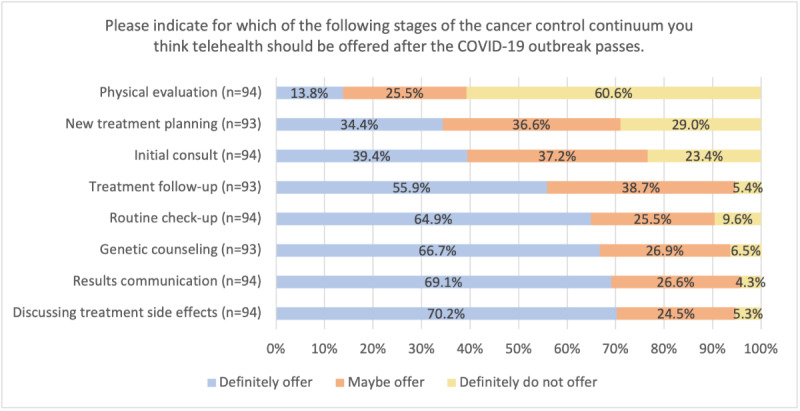
Clinician and staff perceptions regarding appointment types for which telehealth should be offered.

Clinician and staff survey findings mostly mirrored patients’, but there was recognition of the potential role for telehealth in an even greater range of appointment types. A majority of clinicians and staff felt telehealth should “definitely” be offered for discussing treatment side effects (66/94, 70.2%), results communication (65/94, 69.1%), genetic counseling (63/94, 66.7%), routine check-ups (61/94, 64.9%), and treatment follow-up (52/93, 55.9%), as shown in Figure 2.

In semistructured interviews, clinicians elaborated on the multiple considerations that would determine whether they would offer a telehealth versus in-person appointment. One clinician felt that appointments scheduled to discuss laboratory results were an appropriate opportunity to use telehealth because the primary purpose of such appointments is to support conversation as opposed to gathering more laboratory data or providing other treatment. Similarly, another clinician felt that patients on infusion could be followed adequately via telehealth appointments, provided they are in a stable place with their treatment.

Some clinicians indicated that the appropriateness of telehealth depended on the type of cancer the patient has. A urology oncologist commented that telehealth was appropriate for his patients with prostate cancer, whose treatment comprised only hormone shots every 6 months. A hematologist noted there were varying circumstances in which patients with leukemia required in-person visits; lymph node checks required a physical exam, whereas discussing lab results did not.

Other factors included the complexity of a patient’s care, with one clinician noting that the immediate perioperative period for surgical patients could influence the appropriateness of the modality of care delivery. Further, the stage of a patient’s disease was also an important consideration in the eyes of some clinicians, with advanced-stage disease requiring more aggressive monitoring and treatment. Not surprisingly, a majority of clinicians felt physical exams still required in-person visits. One clinician, for example, noted that an in-person exam would be crucial when determining what type of breast surgery was most appropriate for a patient with breast cancer because the breast size, shape, tumor size, and tumor location all factored into her surgical strategy.

### Theme #2: Suggestions to Enhance Remote Cancer Care in the Future

#### Patients

The Cancer Center dedicated significant resources to patient education and technical assistance during the COVID-19 pandemic to facilitate rapid conversion of patient appointments to telehealth. Surveyed approximately one year after the height of the pandemic, fewer than 20% of patients reported experiencing specific challenges with initiating or preparing for telehealth appointments. Specifically, 18% did not understand the video visit setup process, 19% did not feel it was easy to join the video visit session, and 17% did not have enough technical support. But while the vast majority (88%) of the surveyed patients felt sufficiently prepared for their telehealth appointment, less than a third (31%) of surveyed clinicians and staff agreed or strongly agreed that the Cancer Center did a good job of preparing patients for their first telehealth encounter.

Importantly, in semistructured interviews, patients still offered various suggestions about how telehealth care could be enhanced in the future. One participant recommended improving the quality of educational materials pertaining to telehealth, noting they often assume patients have better digital literacy than they actually do. Another patient felt that in-person education for older patients, particularly when initiating or preparing to use telehealth, would be helpful. One individual, who was initially opposed to having telehealth appointments before the pandemic but became a strong supporter of the technology after completing telehealth appointments during the pandemic, suggested offering testimonials to convince patients who may be apprehensive about trying telehealth.

Other patients indicated that telehealth appointments needed to be offered to patients on a more regular basis, acknowledging that they sometimes ask if telehealth is possible when scheduling.

#### Clinicians and Staff

Administrative leaders, clinicians, and front-line staff offered various suggestions based on the different challenges they faced and the telehealth workarounds they devised. During the early part of the pandemic, clinical and administrative staff were assigned to assist patients with initiating their telehealth appointments. This process involved first logging on through a patient’s portal account, and during this step, some patients discovered problems with their portal login credentials. In such cases, knowing what level of familiarity patients already had with the portal was key to triaging a high volume of technical assistance requests. Other staff suggestions to assist patients with limited digital literacy included establishing “fake appointments” for patients to practice online connections and hiring a dedicated employee like a digital health navigator to provide on-call technical assistance and setup of the online portal.

Whereas front-line staff suggestions tended to focus on digital literacy assistance for patients, clinician, and administrator suggestions tended to focus on the need to enhance workflows and infrastructure to improve privacy, efficiency, and clinician training. Multiple clinicians expressed the need for dedicated physical spaces with sufficient privacy for them to conduct telehealth appointments. From a technical standpoint, some clinicians noted that it would also greatly improve efficiency to implement consenting for surgical procedures and clinical trials via telehealth. Some clinicians felt that additional training was needed, for example, to show patients images over telehealth using the “share screen” function.

Despite the challenges they experienced, a majority of both patients (65.7%) and clinician and staff (76.9%) survey respondents reported that they intended to continue using telehealth in the postpandemic era.

## Discussion

### Principal Findings

In this mixed methods study, patients, clinicians, and staff reported an openness toward expanding telehealth-based cancer care across the cancer control continuum. Participants recognized that some situations still favored in-person visits, including appointments in which a physical exam would be necessary and appointments for patients who required more aggressive surveillance due to the stage of their disease. However, both patients and clinicians agreed that telehealth would be convenient and efficient for appointments to discuss treatment side effects, lab results, and treatment follow-up. Clinicians also noted that genetic counseling and consenting patients for surgery or clinical trials could be effectively performed over telehealth. Participants also offered multiple recommendations for health care systems to establish the infrastructure to support these specific opportunities for telehealth-based cancer care delivery.

### Comparison to Prior Work

Despite the reported willingness of older patients to engage in telehealth, literature indicates that older adults are often offered telehealth appointments and patient portal registration at lower rates than younger patients, partly due to health care clinicians’ assumptions that older patients may be less interested in or capable of using digital health tools [32-34]. Such assumptions can inadvertently limit older patients’ access to telehealth, even though many are both willing and able to engage with these technologies when provided adequate support. Such ageist biases highlight the need for intentional strategies to encourage telehealth use among older adults, ensuring that they are not inadvertently excluded from the potential benefits of the technology. Further, given that approximately 65% of cancer cases occur among patients aged 65 years and older [20,21], addressing such inequities among older patients could expand the role of telehealth in cancer care.

Several strategies to improve digital engagement for older adults may serve to address inequities in telehealth service delivery and outcomes for older adults [22-24]. These include technical modifications in portal interfaces to simplify the sign-in process, changes to size or color of text or icons, and incorporation of more pictures as opposed to text, which may improve user experience and capability [35,36]. Other recommendations include patient portal adoption campaigns targeting older adults, task-specific training, support for proxy users, challenging assumptions about older adults and technology, and alternative workflows to allow for communication between patients and personnel to review personal health information within patient portals [22,37]. Clinical and administrative staff may benefit from having policies and procedural guidance detailing when and for whom telehealth appointments should be offered.

Our findings contribute evidence that educational materials must be tailored to older adults or other patients with lower digital health literacy, such as through in-person educational sessions and testimonials from those who have benefited from telehealth. Clinicians and staff on our study also suggested the idea of establishing “fake appointments” for patients to practice using telehealth interfaces and hiring a dedicated employee such as a digital health navigator for technical assistance. The use of digital navigators—trained individuals who assist patients in accessing and using digital health resources—has been found to be effective in enhancing digital literacy and reducing barriers to telehealth access, especially for socioeconomically disadvantaged populations [38-41]. These programs could be particularly beneficial for older adults with cancer, who may face additional challenges due to their complex care needs.

Increasing the use of telehealth for older adults with cancer has the potential to increase independence, patient satisfaction, and quality of life [13-16,42-44]. Studies have found that older adults who learned to use new technology were over twice as likely to adopt telehealth during the pandemic compared to those who did not [45]. In light of the “gray tsunami” phenomenon, where an increasing number of older adults are anticipated to require health care services, and the overwhelming burden of cancer among patients aged 65 years and older [20,21], it is important to recognize the urgency of addressing digital health literacy to minimize health disparities.

### Strength and Limitations

This study has several limitations that impact the potential transferability of our findings to other settings. First, the sample was drawn from a single Cancer Center, with most participants being White and English-speaking, thereby limiting applicability to more diverse populations. Second, data collection for this study occurred during the latter half of 2021, and as such, should be considered within the context of the COVID-19 pandemic. Third, attitudes toward patient portals and telehealth use were evolving during the pandemic, and remote cancer care delivery was a necessity for some patients with cancer during this time. Fourth, by recruiting participants through the portal to sample patients who could adequately comment on telehealth experiences, we may have introduced a bias toward patients who are already more technologically proficient or feel positively about telehealth. This may have limited insights into the experiences of older adults with lower digital literacy, potentially underrepresenting the experiences of those with significant barriers or negative perceptions. Anticipating this limitation, we purposefully recruited 3 individuals without telehealth experience to participate in an interview; however, the small overall patient sample size may also be a limiting factor. Similarly, by recruiting our patient interview sample from our survey sample, we may have introduced a potential response bias toward the inclusion of individuals who felt strongly toward telehealth (positively or negatively). Our recruitment for clinician and staff interviews could also have introduced bias given our reliance on suggestions by our clinician co-investigators. Strengths of this study include its use of the sociotechnical model as a guiding framework for the instrument design and analysis plan, and our inclusion of both patients and clinicians and staff. Finally, our approach of conducting a small number of targeted, preliminary clinician and staff interviews enabled us to assess the appropriateness and representativeness of our survey question items prior to launching our full surveys with patients and other clinicians and staff.

### Future Directions

If telehealth expands across the cancer control continuum, it will be critical that health care organizations prioritize digital equity and inclusion of populations with lower digital literacy. This includes racial and ethnic minorities and older adults and their care partners, to ensure that the needs of these growing populations are adequately met and to provide a more balanced view of the challenges they may face. Future research would benefit from exploring the relative impacts of various interventions, such as digital navigator programs and tailored educational materials, on telehealth engagement and health outcomes.

### Conclusions

This study contributes new evidence that older patients and their clinical care members welcome the expansion of telehealth use across the cancer control continuum. This is an important finding given the fact that digital health technologies, including but not limited to synchronous telehealth visits, are already integral components of health care delivery. It suggests that, as the population ages, health care systems will have the potential to reach a greater share of their patients by enhancing access to cancer services through telehealth and other digital health technologies.

## Supplementary material

10.2196/73058 Multimedia Appendix 1 Survey recruitment consort diagram.

10.2196/73058 Multimedia Appendix 2 Illustrative quotes from patients, clinicians, and staff.

10.2196/73058 Checklist 1 GRAMMS checklist.

## Abbreviations

NCATS: National Center for Advancing Translational Sciences
REDCap: Research Electronic Data Capture
