# Supplementary figures and images for: Patient and Clinician Perspectives on Expanding Telehealth Use for Older Adults Across the Cancer Control Continuum: Mixed Methods Study
Source: JMIR Cancer. 2026 Feb 9;12:e73058. doi: 10.2196/73058 (PMC12885455; doi:10.2196/73058)

## Supplemental Material 1: Survey Recruitment Consort Diagram

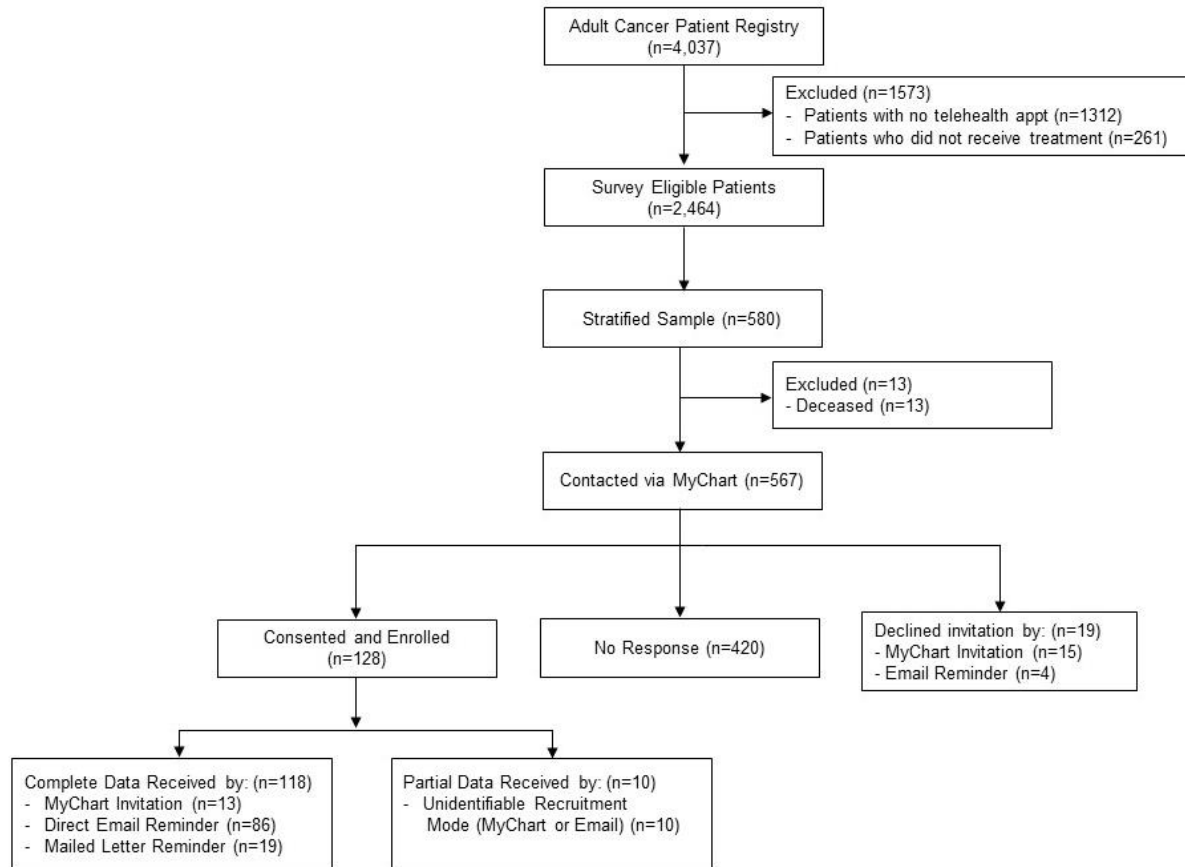

Supplement: Multimedia Appendix 1 [file cancer-v12-e73058-s001.pdf]
