# Supplementary material for: Patient and Clinician Perspectives on Expanding Telehealth Use for Older Adults Across the Cancer Control Continuum: Mixed Methods Study
Source: JMIR Cancer. 2026 Feb 9;12:e73058. doi: 10.2196/73058 (PMC12885455; doi:10.2196/73058)
Supplement: Multimedia Appendix 2 [file cancer-v12-e73058-s002.docx]

| ***Theme #1: Perceptions toward expanding telehealth to other phases of the cancer care continuum*** | | |
| --- | --- | --- |
| *a) Patient perceptions* | Ideal types of telehealth appoint-  ments | “If it’s just after you got out of the hospital…if it’s just follow-up, how are you feeling? Are you taking your medications? Do you have any questions about any of your medications, about your course of care? I think if it was just kind of routine, that kind of follow-up, telehealth would be fine.” – patient with breast cancer (B1E) |
|  |  | “Telehealth is best for appointments where you’re engaged in education, you’re engaged in questions and conversation about the particular situation and the case, what to do next, and the types of things that your care is being based upon or things like lab work and x-rays and MRIs and things that you can get reports on in MyChart.” - patient with prostate cancer (P2E) |
|  |  | “Consent to do the next treatment and set everything up - I already know what is going on with me at that point. I know pretty much what the process is going to be. Now, if it is going to be the very first time, if I was a first-time patient, I’d probably want to do it in person. But if I’d already had the experience and I knew what was going to be happening, doing it virtually is just fine.” – patient with colorectal cancer (C1E) |
|  |  | “When I’m really fatigued or I’m feeling this or that, I could handle that through [the portal] and then we could follow up with a telehealth and the doctor would say, ‘well I want you to get this test or that test and go do that.’” – patient with prostate cancer (P2E) |
|  | Ideal telehealth candidate | “It’s good for mothers with small children that don’t have to load up their kids. I know I have a granddaughter that’s got three small children, and it’s hard for her to go to the doctor, so telehealth would be good.” – patient with breast cancer (B1E) |
|  |  | “From a pure convenience perspective, the perfect telehealth [candidate] is one who has a very difficult time with transportation. And let’s face it, we live in Texas, and we don't have any kind of mass transportation that’s easy.” – patient with breast cancer (B4E) |
| *b) Clinician and staff perceptions* | Ideal types of telehealth appoint-  ments | “If you consider the immediate perioperative period, I think that the low complexity surgeries could be done by telehealth. For my big operations, I’d rather see the patient in person.” - colorectal surgical oncologist (PR20) |
|  |  | “If it’s a really early-stage disease… much of that monitoring can be done by telehealth, whereas if they have more advanced stage disease, then they require more aggressive monitoring and treatment.” – hematology medical oncologist (PR11) |
|  |  | “It is also specialty dependent, so for example…some of my prostate cancer patients are only on hormone shots every six months, [so] I can totally see them as a telehealth [appointment]. So, it’s also disease specific.” – urology medical oncologist (PR12) |
|  |  | “The physical exam is really important in terms of what kind of surgery I offer because the breast size, the breast shape, how big the tumor is, where it is on the skin or close to the skin, or how big their breast is - all those things basically help me figure out what kind of surgery I would offer them or what would be best for them.” – breast surgical oncologist (PR01) |
|  | Ideal telehealth candidate | “Suppose [a patient] just had labs locally, and we know that they don’t need any transfusions, but we just want to talk to them and reassure them that they’re okay and there’s no intervention needed. A telehealth might suffice in a situation like that.” - hematology medical oncologist (PR06) |
|  |  | “I think it’s really the…patients who are stable and doing well on their infusion visits, and they really don’t necessarily need to see the doctor every time they get an infusion visit.” - breast medical oncologist (PR02) |
|  |  | “We have patients who are fresh out of a bone marrow transplant or fresh out of treatment for their leukemia, and we have them come twice a week for lab checks. Now we can make one of those visits tele[health] because we have seen them that week and, unless there is a major change in their symptomatology, I don’t think we physically need to lay hands on them.” – hematology medical oncologist (PR06) |
| ***Theme #2: Suggestions to enhance remote cancer care in the future*** | | |
| *a) Patient suggestions* | Patient-level changes | “Sometimes when you get instructions, they’re not specific enough. They just assume you have a lot more computer knowledge than you do.” – patient with multiple cancers (M2E) |
|  |  | “Schedule [classes] where they can come in and have someone walk them through how to do it and they can make notes or step-by-step instructions and help them set up their device because you’re gonna have a lot of elderly people who can’t do that.” – patient with colorectal cancer (C2E) |
|  |  | “Maybe have some testimonial-type situations with some folks that tell them, ‘I felt very comfortable.’ You know, give them some feedback from other true cases that, ‘I was apprehensive, I wasn’t sure, it went great’… Perhaps present the fact that this is a much better scenario than having to get in your car and go down there or get a ride or have somebody bring you to the office.” – patient with hematological cancer (H3E) |
|  |  | “When an appointment is being scheduled, [give] patients the option – ‘do you want an in-person visit or telehealth visit?’” – patient with breast cancer (B3E) |
| *b) Clinician and staff suggestions* | System-level changes | “Whoever is making the [telehealth] appointment, whether scheduler or intake, if they can document that the instructions for telehealth were given to the patient, that the patient understands how to log on, that would [be helpful]. It wouldn’t be anything real long, but just something that they’ve talked to the patient already.” – nurse (PR14) |
|  |  | “Obviously, these [spaces] need to have the privacy for the patient to feel like nobody’s walking right behind me. It’s hard - you’re talking to somebody and they’re showing something on the video visit. They want to feel that this is a completely private conversation. So having telehealth pods with the required privacy will be nice.” – hematology medical oncologist (PR05) |
|  |  | “Let’s just say our clinic is full and I’ve got six telehealth visits this day. I’ll see a patient in the clinic and then I have to run up and do my telehealth visit, which I am more likely to do up in my office, and then I come back down and see the patients again, and then I run up to the office. There is the option to do it in these clinic spaces, but there’s other people around, so it’s not really private. So, [I’m] just sort of bouncing back and forth.” – prostate medical oncologist (PR12) |
|  |  | “Well, I want us to figure out consenting through telehealth because I think that helps us with clinical trials, but it could also help us with just expedition of getting people ready for surgery.” – breast surgical oncologist (PR13) |
|  |  | “[I wish] we as physicians could say, ‘here’s the scan that we’re all looking at’. Right now, I don’t think that you can do that through BlueJeans, or if you can, I’m not savvy enough with the technology to do it. But in oncology, simply having the opportunity to look at a scan with a patient is a useful tool.” – prostate medical oncologist (PR16) |
