# Supplementary material for: Patient and Clinician Perspectives on Expanding Telehealth Use for Older Adults Across the Cancer Control Continuum: Mixed Methods Study
Source: JMIR Cancer. 2026 Feb 9;12:e73058. doi: 10.2196/73058 (PMC12885455; doi:10.2196/73058)
Supplement: Checklist 1 [file cancer-v12-e73058-s003.docx]

Appendix 3. Good Reporting of A Mixed Methods Study (GRAMMS) Checklist

| Guideline | Section/Page  *(note: page numbers correspond to viewing the manuscript WITHOUT track changes)* |
| --- | --- |
| Guideline Justification to use a mixed methods approach to the research question | Study Design, pp. 5-6 |
| Articulation of the design in terms of purpose, priority, and sequence of methods | Study Design, pp. 6-7 and Data Collection, p. 9 |
| Describe each method in terms of sampling, data collection and analysis | Sampling, pp. 6-7; Data Collection, p. 9, Data Analysis, pp. 9-10 |
| Delineate where and how integration occurs and who has participated in it | End of 1^st^ paragraph of Data Analysis, pp. 9-10 |
| Describe any limitation of one method associated with the presence of another | Strengths and Limitations section, pp. 19-20 |
| Describe insights gained from mixing or integrating methods | Strengths and Limitations section, pp. 19-20 |

O'Cathain A, Murphy E, Nicholl J. The quality of mixed methods studies in health services research. J Health Serv Res Policy. 2008;13: 92-98.
